# Abuse of Medicine

**Published:** 1859-12

**Authors:** 


### ABUSE OF MEDICINE.

" I sincerely believe that the unbiassed opinion of most medical men of sound judgment and long experience is made up, that the amount of death and disasters in the world would be less if all disease were left to itself, than it now is under the multiform, reckless, and contradictory modes of practice, good and bad, with which practitioners carry on their differences at the expense of their patients."

Such is the declaration of a Boston physician, who has published a book. We will venture the assertion that " most medical men of sound judgment and long experience," will give it as their " unbiassed opinion," that the writer of the above has maligned his brethren, and has degraded himself by uttering a sentiment so unjust and so foreign to the truth.

Our own experience is that the longer we live, the more fully are we convinced of the value of medicine as a remedial agent in disease; and such we will venture to say is the experience of every practical physician in the land, of education, and of skill. And such we think is the opinion to-day, of Dr. Jacob Bigelow, of Boston town, of the noble old Bay State. In justice to the doctor, we are compelled to believe, that in writing the above sentiment, he got in a fog, and kept on writing, in the hope of seeing daylight, but failed to make his point, and closed in a kind of desperation, without knowing exactly what he did say, as the unregenerated lawyer who, on being unexpectedly asked to return thanks after dinner, couldn't exactly get at the "Amen," but ended by a "Respectfully, Yours."

The abuse of medicine by the ignorant and unprincipled, more than counterbalances its good effects in the hands of educated and skillful practitioners, is what the Doctor intended to say, but from some cause or other, he could'nt bring his mind to a focus. But even that statement is foreign to the truth in our opinion, for by the light which medical men have thrown out in their investigations as to health and disease, the average duration of human life has travelled up from twenty-one years in the sixteenth century, to forty-one in the nineteenth; or expressing it in another form, a child born at Geneva in the sixteenth century, would probably live five years, but born at the present moment, he would, with equal probability, live forty-four years. All honor then to the Profession, to the Art, to the Science, which in three centuries has by its laborious investigations, and by the liberal diffusion of its light, largely aided to increase the duration of human life nearly nine times.

